# Effects of Music on Cardiovascular Responses in Men with Essential Hypertension Compared with Healthy Men Based on Introversion and Extraversion

**DOI:** 10.15171/jcvtr.2014.009

**Published:** 2014-09-30

**Authors:** Hossein Namdar, Mohammadreza Taban Sadeghi, Hassan Sabourimoghaddam, Babak Sadeghi, Davoud Ezzati

**Affiliations:** ^1^Cardiovascular Research Center, Tabriz University of Medical Sciences, Tabriz, Iran; ^2^Department of Psychology, Faculty of Educational Sciences and Psychology, University of Tabriz, Iran

**Keywords:** Music, Essential Hypertension, Introversion, Extraversion

## Abstract

*Introduction:* The present research investigated the effects of two different types of music on cardiovascular responses in essential hypertensive men in comparison with healthy men based on introversion and extraversion.

*Methods:* One hundred and thirteen hypertensive men referred to Madani Heart Hospital in Tabriz completed the NEO-FFI Questionnaire and after obtaining acceptable scores were classified in four groups: introvert patients, extravert patients, introvert healthy subjects, and extravert healthy subjects (each group with 25 samples with age range 31-50). Baseline blood pressure and heart rate of each subject was recorded without any stimulus. Then subjects were exposed to slow-beat music and blood pressure and heart rate were recorded. After15 minute break, and a little cognitive task for distraction, subjects were exposed to fast-beat music and blood pressure and heart rate were recorded again.

*Results:* Multivariate analysis of covariance (MANCOVA) test showed that extravert patient subjects obtained greater reduction in systolic blood pressure and heart rate after presenting slow-beat music compared with introvert patients (P= 0.035, and P= 0.033 respectively). And extravert healthy subjects obtained greater reduction in heart rate after presenting slow-beat music compared with introvert healthy subjects (P= 0.036). However, there are no significant differences between introvert and extravert groups in systolic and diastolic blood pressure and heart rate after presenting fast-beat music.

*Conclusion:* Based on our results, introvert subjects experience negative emotions more than extravert subjects and negative emotions cause less change in blood pressure in these subjects compared with extravert subjects.

## Introduction

Cardiovascular diseases remain the most common cause of death in the world. According to the World Health Organization (WHO) three in every ten deaths in 2011 were due to cardiovascular diseases.^1^ Because of inducing physical and psychological stressors, cardiovascular diseases cause feelings of worthlessness and loss of confidence in the patient.^2^

Hypertension is a phenomenon that has an important role in cardiovascular diseases.^3-5^ This phenomenon is one of the public concerns, and its prevalence is increasing globally, especially in developed countries.^6^ The WHO has estimated that out of every eight deaths worldwide, one is caused by hypertension, making hypertension the world’s third leading cause of death.^7^

Many studies have investigated the effects of psychological variables on physiological indices.^8-11^ Introversion/extraversion is a dimension with a range between two extremes, and within this range, there are people that are neither extravert nor introvert. About the difference between introversion and extraversion, Eysenck suggests the concept that extraverts have fairly strong inhibitory processes, but poor excitability. Additionally, with robust nervous systems, they have a high capacity for accepting stimulation. Hence, extraverts’ brains react to stimuli more slowly and weakly, leading to inclination toward strong sensory stimulation. Introverts have strong stimulatory processes. In terms of the cortex, they are inherently more motivated, with brains that react faster and more strongly to stimuli, and have a low threshold for stimulation.^12-14^ The inhibition theory leads to a series of behavior predictions that have generally been experimentally confirmed. For example, extraverts are interested in listening to loud music, bright colors, smoking, and drinking.^15^

Music plays an important role in social and working lives of the young and the old alike.^16-18^ Mood swings such as happiness, sorrow, anxiety, and anger may also be associated with change in physiological indices.^19^ Since results obtained about impact of mood on cardiovascular responses are inconsistent, it can be assumed that personal differences in other areas like personal characteristics could probably modulate the association of mood states with cardiovascular responses.

## Materials and methods

This study is a quasi-experimental research. Study subjects consisted of 211, 30- to 50-year-old men including 113 male patients with essential hypertension confirmed by cardiologist, and 98 healthy men, matched with the patients for demographic variables. After completing the revised short form of NEO-FFI, and gaining an acceptable score (scores of 12 to 24 for introversion dimension and 48 to 60 for the extraversion dimension), the patients and healthy subjects were divided into two groups based on the introversion and extraversion. Each group consisted of 25 participants, totaling 100. In cases where the subjects were not able to understand the concepts, the researcher was clarifying with more information. Convenience sampling was used in this study. Those patients with arrhythmias were excluded from the study. Prior to completing forms, participants were informed of study objectives and explanations were given about the questions and that they could withdraw at any time they wished. Mood was created through slow-beat music (a piece by Mohammad Naeim titled Farah), and fast-beat music (a piece by Yanni, titled York-the awakening). Subjects were asked to be in a silent room and seated position to listen to the music using headset with same volume for both types of music. Before playing the music pieces, systolic and diastolic blood pressures and heart rate of participants were measured.

Slow-beat music was played for three minutes, and afterward, their blood pressure and heart rate were measured. Between the two types of music, to eliminate confounding effects, participants rested for 15 minutes. During this time, they carried out a cognitive task (subtracting 7s from 100). Then, fast-beat music was played for three minutes, and again blood pressure and heart rate were measured.

### Study tools included

1. The revised NEO-FFI questionnaire (short form), which contains 60 items and measures five personality traits: neuroticism, extraversion, agreeableness, openness, and conscientiousness. This tools is answered with the 5-point Likert scale (from totally agree to totally disagree). Every personality trait is assessed by 12 items. The content validity of the questionnaire was found by McCrae & Costa, and its reliability for personality trait of neuroticism was reported 90%, for extraversion 78%, for openness 76%, for agreeableness 86%, and for conscientiousness 90%.^20^
2. The digital arm sphygmomanometer: Participants’ systolic and diastolic blood pressures and heart rate were measured before playing the music and also immediately after induction using the digital arm sphygmomanometer.
3. Headset device: Both types of music were played for the participants using this device.

### Analysis of data

Central tendency indicators (frequency, mean, and standard deviation) were used in descriptive statistics. Multivariate covariance analysis (MANCOVA) test were used in inferential statistics for examining study question. Also, to ensure data normality and to comply with conditions of use of parametric statistics, Kolmogorov-Smirnov test was used. BOX test was used to verify equality of covariance parameters assumption, and levene’s test was used to examine assumption of equality of groups’ co-variances. To ascertain presence of a linear relationship between pretest and post-test scores, the regression slope test was used. *P* values less than 0.05 were considered statistically significant.

## Results

Kolmogorov-Smirnov test revealed normal distribution of data (P>0.05). BOX test confirmed equality of covariance parameters, and their insignificant differences (P>0.05). Levene’s test showed equality of study variables variance, and their insignificant differences (P>0.05). Results of the regression slope test showed that there was no significant interaction between group and pre-test scores in none of the physiological indicators (systolic blood pressure, diastolic blood pressure, and heart rate). In other words, regression slopes are homogeneous, and assumption of regression slopes was confirmed.

Table 1, relates to the descriptive statistics of the four participating groups, indicating that in both stages (pre-test and post-test), extravert compared to introvert patients, and healthy extraverts compared to healthy introverts had higher reduction in cardiovascular responses. The covariance analysis results of groups’ cardiovascular responses showed that among cardiovascular response scores of four groups, a significant difference was observed in systolic pressure and heart rate after playing slow-beat music and systolic pressure after playing fast-beat music (P<0.001, P<0.001, and P<0.001 respectively). There were not any significant differences in diastolic blood pressure after playing slow-beat music and fast-beat music and in heart rate after playing fast-beat music. Table 2 presents comparison of four groups’ mean differences, in which systolic pressure and heart rate in extravert patients compared to introvert patients significantly reduced after playing slow-beat music (P=0.035, and P=0.033 respectively). Also, heart rates of healthy extraverts compared to introverts significantly reduced after playing slow-beat music (P= 0.036). However, no significant differences were found in other cases.

**Table 1:** Mean and Standard deviation of systolic and diastolic blood pressure and heart rate

**Dependent variable**	**Introvert**			**Extravert**		
	**Before stimulus** **Mean ± SD**	**After slow-beat music** **Mean ± SD**	**After fast-beat music** **Mean ± SD**	**Before stimulus** **Mean ± SD**	**After slow-beat music** **Mean ± SD**	**After fast-beat music** **Mean ± SD**
Patients						
**** SBP	146.8 ± 4.9	139.9 ± 5.3	141.6 ± 6.0	135.9 ± 2.5	128 ± 3.9	128.8 ± 4.9
DBP	90.0 ± 4.8	89.8 ± 5.0	89.3 ± 3.6	87.3 ± 5.1	84.2 ± 5.2	86.32 ± 3.6
HR	90.2 ± 8.8	89.4 ± 2.6	87.8 ± 5.3	83.4 ± 3.0	71.5 ± 4.3	76.9 ± 4.4
Healthy subjects						
SBP	117.0 ± 5.2	108.1 ± 3.9	114.4 ± 4.2	108.2 ± 5.5	103.6 ± 7.2	109.4 ± 5.2
DBP	72.3 ± 3.8	71.6 ± 5.6	76.5 ± 8.1	69.6 ± 7.9	70.1 ± 7.3	71.6 ± 10.4
HR	86.16 ± 12.5	84.2 ± 7.2	84.1 ± 9.2	79.6 ± 9.9	71.4 ± 3.8	77.1 ± 9.0

SBP, Systolic Blood Pressure; DBP, Diastolic Blood Pressure; HR, Heart Rate Introvert patients (N=25); Extravert patients (N=25); Introvert healthy subjects (N=25); Extravert healthy subjects (N=25)

**Table 2:** Paired comparison between introvert and extravert groups in SBP, DBP, and HR

**Changes in dependent variable**	**Patients**			**Healthy subjects**		
	**Mean difference**	**Std. Error**	P*	**Mean difference**	**Std. Error**	P*
After slow- beat music						
**** SBP	13.685	6.26	0.035	5.678	4.774	0.243
DBP	4.974	8.787	0.568	-6.657	4.526	0.176
HR	17.613	8.136	0.033	14.214	7.115	0.036
After fast-beat music						
SBP	3.889	6.354	0.557	-2.234	6.616	0.748
DBP	-6.029	9.344	0.524	-7.298	5.275	0.094
HR	1.866	9.698	0.84	8.618	5.674	0.145

SBP, Systolic Blood Pressure; DBP, Diastolic Blood Pressure; HR, Heart Rate *P<0.05

## Discussion

In this study, extravert patients compared to introvert patients showed higher reduction in systolic blood pressure and heart rate after hearing soft music. Also, healthy extravert people compared to healthy introvert people showed higher reduction in heart rate after hearing soft music.

According to Eysenck theory, extravert people compared to introvert people showed more sensitivity to positive emotional stimulus. Thus, if this theory is right, extraverts compared to introverts are more responsive to positive emotional induction.^21^ Also, Gray in his theory, points out high sensitivity of Behavioral Approach System (BAS) people to rewarding signs, which somehow reflects extraverts’ early arousal component.^22^

Harvey and Hirshman showed the moderating role of personality dimensions of extraversion and introversion in heart rate changes in response to aversive stimuli.^23^ In another research, Rafieinia et al. showed that in positive mood situations, extraverts compared to introverts (following music) had more reduction in systolic blood pressure, and in negative mood situations, heart rate of introvert people compared to extraverts had increased.^24^ De Pascalis et al. found different sensitivities of cardiovascular responses of extraverts and introverts to reward and punishment signs.^25^ Also, in relation to the moderating role of personality traits, studies by Hamer et al. could be cited, which reveal personality traits and fundamental characteristics significantly influence impacts of emotional stimuli on cardiovascular responses.^26^ According to results of a study by Yu et al. mood state of boredom is associated with main factors affecting blood pressure through sympathetic activity.^19^ There is also evidence that shows among the negative emotions, anger and worry have the strongest association with hypertension.^27^ Fredrickson et al. and Iribarren et al. argue that high levels of negative emotions are associated with higher reactivity of blood pressure to psychological pressure.^28,29^

Nevertheless, studies by Bartusske et al. did not show any distinct readiness in introverts and extraverts toward positive and negative stimuli.^30^ Also, Russo and Zuckerman showed that extraverts and sensation seekers’ blood pressure was higher compared to people with different personalities.^31^

## Study limitations and recommendations

The present study had limitations that affect generalizability of its results. Firstly, the study was conducted only on the 30- to 50-year-old. Secondly, participants were selected from male population only, and finally, because the follow up of all patents were not possible, cross over design with switching the order of the music was not performed in this study.

## Conclusion

In connection, with Eysenck’s theory, since introvert people experience more negative emotions due to sensitivity to punishment and also overactive inhibitory system, and extravert people compared to introvert people are more sensitive to reward, it could be asserted that the results obtained about differences between these groups appear to be logical, as such, introvert patients showed less decrease in cardiovascular responses compared to extravert people. Therefore, introvert patients could use psychiatric counseling for better control of hypertension and prevention of blood pressure fluctuations during the day in addition to medication treatment.

## Ethical issues

This study was reviewed and confirmed by the ethics committee of Tabriz University of Medical Sciences.

## Conflict of interests

The authors declare no conflict of interest in this study.
